# Resilience Against Traumatic Stress: Current Developments and Future Directions

**DOI:** 10.3389/fpsyt.2018.00676

**Published:** 2018-12-14

**Authors:** Clara Snijders, Lotta-Katrin Pries, Noemi Sgammeglia, Ghazi Al Jowf, Nagy A. Youssef, Laurence de Nijs, Sinan Guloksuz, Bart P. F. Rutten

**Affiliations:** ^1^Department of Psychiatry and Neuropsychology, Faculty of Health, Medicine and Life Sciences, School for Mental Health and Neuroscience (MHeNS), Maastricht University, Maastricht, Netherlands; ^2^College of Applied Medical Sciences, Department of Public Health, King Faisal University, Al-Ahsa, Saudi Arabia; ^3^European Graduate School of Neuroscience, Maastricht University, Maastricht, Netherlands; ^4^Department of Psychiatry and Health Behavior, Medical College of Georgia, Augusta University, Augusta, GA, United States; ^5^Office of Academic Affairs, Medical College of Georgia at Augusta University, Augusta, GA, United States; ^6^Department of Psychiatry, Yale School of Medicine, New Haven, CT, United States

**Keywords:** resilience, stress, prospective longitudinal studies, resilience-promoting interventions, review

## Abstract

Given the high prevalence of stress-related mental disorders, their impact on person, family, and society and the paucity of treatment options for most of these disorders, there is currently a pressing need for innovative approaches to deal with these issues and enhance well-being. One approach which has received increasing attention over the last decade is to shift our scientific and clinical focus from risk factors for psychopathology to factors promoting resilience and mental well-being. In order to summarize and evaluate the current state of scientific affairs on the biological basis of resilience, we provide an overview of the literature on animal and human studies of resilience. Because resilience can only truly be operationalized through longitudinal data collection and analyses, we focus primarily on longitudinal studies. This review shows that the concept of resilience is currently being operationalized, measured and even defined in widely variable manners, both within animal and human studies. We further provide an overview of existing and new strategies that could help promote resilience and which are proposed to be implemented more often in clinical situations. Finally, we summarize the challenges the field is facing and provide recommendations for future research.

## Introduction

Over the past decade, research on resilience has received increasing attention. The heightened interest in understanding and promoting resilience is not surprising given that in Europe alone, anxiety disorders and major depressive disorder were among the most frequent mental disorders in 2011 with a 12-month prevalence of 14% (corresponding to 61.5 million individuals) and 6.9% (30.3 million), respectively (1). This is not much different than the prevalence of these disorders in North America and many other parts of the world. Moreover, a recent meta-analysis showed that relapse rates in patients suffering from depression remain high and that long-term effects of conventional treatment options are not always encouraging (2). These findings call for additional strategies and alternative interventions needed to prevent disease development and boost resilience.

Here, we conceptualize resilience as being an active and dynamic process through which a person adaptively overcomes a stressful or difficult situation or recovers swiftly from a period of ill-health (3). Thus, resilience is not a passive reaction to an adverse situation, nor is it merely the reverse side of post-traumatic stress disorder (PTSD) or the absence of symptomatology (3, 4). Research on resilience is facing several challenges of which the most fundamental one consists of the enormous heterogeneity in defining and operationalizing resilience. Indeed, some researchers will conceptualize resilience as being a personality trait while others will use the terms outcome, coping strategy or dynamic process (see 5) for a critical overview of different theories on resilience). Such discrepancies impede its measurement and further hinder the comparison of obtained results across resilience studies. Therefore, the following proposals have recently been formulated to guide future resilience research; (i) the fast recovery of mental health following stress exposure reflects a dynamic adaptation process, (ii) resilience should not be understood as a personality trait, the result of a specific genotype or other hardwired characteristics, (iii) there is a strong need for prospective longitudinal studies, and (iv) resilience can only be operationalized following a stressful period or event (3). Such stressors can refer to various types of (potentially) traumatic or life-threatening events, injury, illness, or difficult life circumstances such as unemployment, grief or divorce. Although most individuals recover promptly following trauma exposure, gaining a deeper understanding of the mechanisms underlying resilience could be of use to those more at risk of developing a stress-related disorder.

Following the conceptualization of resilience as proposed by Kalisch et al. (3), the main aim of the present review is to summarize the findings from key longitudinal animal and human studies on resilience. Furthermore, we propose strategies aiming to promote resilience, discuss limitations and challenges of current resilience research, and suggest future directions to help this field evolve.

## Resilience Studies

Over the past years, most resilience studies used cross-sectional designs. However, susceptibility and resilience to past or ongoing stress are hard to capture when assessing mental health at one time point only. Moreover, these designs do not allow for the exclusion of baseline differences between subjects which further impairs obtaining a comprehensive interpretation of the obtained findings. Using a longitudinal design enables one to assess dynamic behavioral and biological fluctuations over time, enabling investigations of (baseline) predictors of differential susceptibility to future stress. This is highly relevant, especially in light of at-risk jobs where trauma exposure is more prevalent. In order to take this into account, the first part of this review only includes prospective longitudinal animal and human studies, i.e., studies in which the individuals' mental health or the animals' behavioral or physiological state were quantitatively assessed before the (natural or experimental) stress exposure and at least once after this period. Expanding on the review by Kalisch et al. (3), the literature was searched for studies published in 2017 which fulfilled several criteria. First, we only included studies in which the severity and duration of the stress exposure was precisely quantified. For the human studies specifically, we only included studies in which the baseline assessments of predictors of mental health were recorded. In addition, and as postulated by Kalisch et al. (3), in susceptible individuals, the severity of the stress exposure had to positively correlate with the development of mental health problems. Studies were also only considered when including adults and the study groups consisted of 30 subjects or more (3). Since stress responses in children vary depending on their developmental stage, discussing these findings would require a different focus which does not fit within the scope of the present review. Finally, it needs to be noted that no systematic search of the literature was performed given the wide variety of terms used by researchers to describe resilience.

## Animal Studies

In animal research, resilient phenotypes are often identified based on specific behavioral outcomes following a well-defined period of experimentally induced stress. Specifically, those animals showing a fast recovery from a stressful manipulation are said to be resilient. Although caution and critical evaluation of the observed phenotypes are needed when interpreting and translating the obtained findings, these models can provide us with some of the molecular underpinnings of differential susceptibility to stress. Using the above-mentioned eligibility criteria, we identified six studies which can be found in Table 1. Studies sharing similar features are discussed together in the next paragraphs.

**Table 1:** Longitudinal animal studies assessing biological outcomes associated with differential susceptibility to stress.

**References**	**Species and sex**	**Stressor**	**Outcome of interest**	**Main finding**
Chen et al. (6)	Male Sprague–Dawley rats	7 days of chronic social defeat stress	Circulating miRNAs (tail blood)	↓ Pre-stress miR-24-2-5p, miR-27a-3p, miR-30e-5p, miR-362-3p, associates with future vulnerability to chronic social stress. ↓ Post-stress miR-139-5p, miR-28-3p, miR-326-3p, miR-99b-5p associates with ongoing resilience.
Hodes et al. (7)	Male CD45.1+/CD 45.2+ C57BL/6 mice	10 days of repeated social defeat stress	Blood leukocytes and IL-6 levels	Higher pre-stress leukocyte levels in mice that later became stress vulnerable. Higher IL-6 levels following acute stress, only in those mice that later became stress vulnerable.
Kim et al. (8)	Male C57BL/6N mice	Chronic restraint stress	Plasma corticosterone	Longitudinal changes in corticosterone reflect differential stress susceptibility and pre-stress corticosterone predicts post-stress susceptibility or resilience.
Magalhaes et al. (9)	Male Wistar rats	3 weeks of chronic unpredictable stress	Neuroimaging—functional connectivity and structural changes	Pre-stress differences in functional connectivity in brainstem-limbic area between susceptible and resilient rats.
Rasmussen et al. (10)	Male Wistar rats	Inescapable footshock + weekly 1-min reminders for 6 weeks	Acoustic startle response	↑ Pre-stress acoustic startle response = ↑ post-stress plasma corticosterone levels and ↑ post-stress acoustic startle response.
Tse et al. (11)	Male C57BL/6 mice	10 days of chronic social defeat stress	Hippocampal volume	↑ Post-stress left hippocampal volume in resilient and control mice.

*Studies were listed in alphabetical order based on the surname of the first author. miRNAs: microRNAs. IL-6: interleukin-6*.

### Inflammatory Markers

One study examined acoustic startle responses (which has been related to PTSD) in rats before and after exposure to a single episode of inescapable footshocks and 1-min reminders for the next 6 weeks (10). Rats exhibiting a high baseline startle response showed a significantly higher startle response following the period of exposure. Interestingly, rats showing an increased startle response at baseline also exhibited elevated plasma corticosterone levels at follow-up as compared to the rats with low baseline startle responses (although it should be noted that corticosterone levels were not measured prior to the experimental manipulation). Links between increased corticosterone levels and stress-induced behavioral phenotypes have also been observed in other studies (8, 9). One of these studies investigated whether longitudinal changes in blood corticosterone levels were associated with measures of differential susceptibility to stress in mice (8). The authors found that mice with an increase in plasma corticosterone levels upon 2 weeks of repeated restraint stress exposure showed significant weight loss over the course of the experiment as well as anxiety-related behaviors at follow-up as measured through the Elevated Plus Maze (EPM) and Open Field Test (OFT). These mice were thus characterized as being susceptible while more resilient mice were identified when showing (i) a decrease in corticosterone levels from baseline to follow-up, (ii) a stable body weight, and (iii) no anxiety-related behavior. Furthermore, the authors found that corticosterone levels at baseline predicted the extent of change in corticosterone levels during stress exposure and correlated with behavioral measures at follow-up. Another study used a repeated social defeat stress paradigm of 10 days and showed that those mice that later became susceptible to stress exposure had higher baseline levels of leukocytes (7). Moreover, in response to acute stress and prior to the repeated stress exposure paradigm, the same mice exhibited higher levels of the pro-inflammatory cytokine interleukin-6 (IL-6). Together, these results suggest that baseline levels of specific inflammatory markers might predict differential susceptibility to future stress exposure in mice.

### MicroRNAs

Only one prospective study examined the potential of microRNAs (miRNAs) to distinguish resilient and vulnerable animals (6). miRNAs are small, non-coding RNAs which are involved in the post-transcriptional regulation of gene expression (12). miRNAs have been widely studied in cancer and cardiovascular disease as potential biomarkers, but less is known regarding their involvement in mental disorders. (6) examined whether miRNAs could serve as biomarkers of resilience or vulnerability to stress by using a chronic social defeat paradigm in rats that lasted for 7 days. Rats showing little or no avoidance behavior when encountering an unfamiliar rat in its own cage were identified as being resilient while susceptible rats spent less time interacting with the novel rat as compared to controls. The authors found that susceptible rats had lower baseline blood circulating levels of miR-24-2-5p, miR-27a-3p, miR-30e-5p, and miR-362-3p compared to unexposed controls. However, the more resilient rats had lower levels of blood circulating levels of miR-139-5p, miR-28-3p, miR-326-3p, miR-99b-5p at follow-up as compared to controls. At both time points, no differences in miRNA expression were found between resilient and susceptible animals. These results pinpoint a number of candidate microRNA species which could, at least in part, regulate vulnerability to future stress or reflect ongoing resilience to chronic social stress in rats.

### Neuroimaging Data

Using magnetic resonance imaging (MRI) in mice, Tse et al. were the first to assess changes in hippocampal volume prior to and following stress exposure (11). The authors identified susceptible and resilient animals based on their behavioral profiles in the social defeat paradigm. Following stress exposure, approximately half of the mice were classified as susceptible to stress, while the other half was more resilient. In contrast to the susceptible mice which showed no hippocampal volume increase over time, resilient and non-stressed control mice showed an increase in the left hippocampal volume from baseline to post-stress exposure, suggesting that normal hippocampal growth was impaired in susceptible animals only. Intriguingly, a positive correlation was observed between hippocampal volume at baseline and social avoidance behavior at follow-up. These findings suggest that that differences in hippocampal volumes could be associated with vulnerability to future stress in mice, which is further supported by similar findings in humans (13).

More recently, another study assessed structural changes more broadly along with alterations in the brain's functional connectome upon stress exposure (9). In this study, rats underwent a chronic unpredictable stress protocol for 3 weeks. Blood corticosterone levels, MRI scans and anxiety-related behaviors (measured through the EPM) were measured before, 7 and 21 days after exposure to stress. The authors categorized the stress-exposed rats into susceptible and resilient groups based on aberrant behavior and plasma corticosterone levels. Those rats expressing lower levels of post-exposure corticosterone in combination with minimal anxiety-like behavior were categorized as being resilient. Among a broad variety of structural and functional alterations induced by stress, it was found that baseline differences in functional connectivity measures of a specific brainstem-limbic network were able to distinguish the resilient and susceptible groups, with susceptible rats showing lower functional connectivity compared to the resilient ones. It is worth mentioning that in humans, distinct patterns of brain activity have also been linked to PTSD and treatment response (14–16). Together, these results suggest that imaging data can contribute to a better understanding of the psychopathology of PTSD and potentially serve as a predictive biomarker of future vulnerability to stress and/or treatment response.

## Human Studies

Various collaborative prospective approaches are currently being conducted and followed-up on, including longitudinal approaches such as PRISMO (“Stressgerelateerd Militia Onderzoek”) (17–21), the Marine Resiliency Study (MRS) I and II (22–24), and the prospective-longitudinal component of the Prevalence, Incidence and Determinants of PTSD and Other Mental Disorders (PID-PTSD^+3^) (25, 26). Following our eligibility criteria, 12 articles were identified. All dealt with deployment-related stress, i.e., long-lasting effects of events experienced during actual combat, assault or other living conditions experienced during deployment (3). Studies sharing similar features are discussed together in the following paragraphs and can be found in Table 2.

**Table 2:** Longitudinal human studies assessing biological outcomes associated with differential susceptibility to stress.

**References**	**Sample (N)**	**Main stressor**	**Outcome of interest**	**Main finding**
**GENETIC FACTORS**				
Clark et al. (27)	RINGS; Male soldiers: *N* = 253	Deployment	PTSD	Met/Met and Val/Val genotypes had stronger trauma-responses than the Val/Met genotype.
Wald et al. (28)	Israeli Defense Force; Male soldiers: *N* = 1,085	Deployment	PTSD	Threat bias interacted with combat exposure and threat bias interacted with combat exposure and 5-HTTLPR.
**EPIGENETIC FACTORS**				
Rutten et al. (20)	PRISMO and MRS; Male soldiers/marines: *N* = 93, *N* = 98	Deployment	PTSD	Genome-wide changes at 17 positions and 12 regions were associated with PTSD status.
Schur et al. (21)	PRISMO, Male soldiers: *N* = 92	Deployment	Mental health and PTSD	Pre-deployment GR-1F region (52 loci) methylation did not predict mental health or PTSD status.
Van Zuiden et al. (17)	PRISMO Male soldiers: *N* = 68	Deployment	PTSD	mRNA expression of GR–α, GR-P, GR-β, glucocorticoid- induced leucine zipper (GILZ), serum and glucocorticoid-inducible kinase-1 (SGK-1), or FKBP5 does not predict PTSD status.
**CIRCULATING MARKERS**				
*Inflammatory Markers*				
Breen et al. (23)	MRS II and MRS; Male marines: *N* = 124, *N* = 50	Deployment	PTSD	PTSD status associated with gene co-expression networks related to innate immune responses.
Eraly et al. (22)	MRS, Male marines: *N* = 1,719	Deployment	PTSD	Baseline plasma levels of C-reactive protein (CRP) predicted PTSD symptoms.
Smid et al. (18)	PRISMO, *N* = 693	Deployment	PTSD	Interaction between cytokine production, stress exposure during combat and post-deployment stressful life events.
Torshizi et al. (24)	MRS II and MRS; Male marines: *N* = 124, *N* = 50	Deployment	PTSD	PTSD status associated with gene co-expression network master regulators: SOX3, TNFAIP3, TRAFD1, POU3F3, STAT2, and PML.
*Hormonal Dysregulations*				
Reijnen et al. (19)	PRISMO, Male soldiers: *N* = 907	Deployment	PTSD	No moderating effect of plasma oxytocin and arginine vasopressin on stress-related PTSD development.
Steudte-Schmiedgen et al. (25)	PID-PTSD+3, Male soldiers *N* = 90; *N* = 80	Deployment	PTSD	Decreased baseline hair cortisol and cortisol stress predict higher stress-related PTSD.
Trautmann et al. (26)	PID-PTSD+3, Male soldiers *N* = 153, *N* = 145	Deployment	Alcohol consumption	Decreased baseline hair cortisol stress predict higher stress-related alcohol use.
Van Zuiden et al. (17)	PRISMO, Male soldiers: *N* = 68	Deployment	PTSD	Plasma cortisol does not predict PTSD status.

*MRS, MRS II, Marine Resiliency Study I, II; PID-PTSD^+3^, Incidence and Determinants of PTSD and Other Mental Disorders; PRISMO, Stressgerelateerd Militia Onderzoek; RINGS, The Readiness and Resilience in National Guard Soldiers Study. Within each section, studies were listed in alphabetical order based on the surname of the first author*.

### Genetics

Two studies examined the longitudinal effects of genetic variations on stress-related PTSD symptoms. Both studies focused on candidate genes, i.e., the serotonin transporter (*5-HTT*) gene and catechol-O-methyltransferase (*COMT*) gene, which are known to affect serotonergic and dopaminergic signaling, respectively.

In the first study, the effects of a serotonin transporter gene-linked polymorphic region [5-HTTLPR] and threat-related attention on post-deployment PTSD symptoms was evaluated in 1,085 male soldiers (28). PTSD symptoms and threat-related attention bias (measured with a computerized dot-probe task) were assessed three times with the last assessment taking place around 1 year after baseline. Combat exposure between the baseline and follow-up assessments was inferred by using geo-operational exposure data and self-report measures [i.e., the Combat Experiences Scale with two additional items (29)]. The authors observed that pre-deployment-threat bias interacted with combat exposure during deployment and 5-HTTLPR in predicting post-deployment PTSD symptoms. More specifically, fewer post-deployment PTSD symptoms after high combat exposure were found in those individuals who displayed pre-deployment threat vigilance and had the SS/SL-G alleles (i.e., reflecting low transcription 5-HTT) of the 5-HTTLPR genotypes. This study is particularly interesting in highlighting the complex interaction between context, stress-exposure, attention bias and genetics, suggesting that serotonergic transmission may be involved in the co-occurrence of avoidance and hypervigilance symptoms in PTSD (30).

In another study, 253 Iraq war veterans were assessed prior to and following a deployment period of 16 months (27). Deployment trauma was measured with the Post Deployment Stressors subscale of the Deployment Risk and Resilience Inventory (DRRI) (31) as well as by using one additional item on sexual assault experienced during deployment. DNA was extracted from blood or buccal swabs and was genotyped into COMT Met/Met (*n* = 63), Val/Met (*n* = 131), or Val/Val (*n* = 42). Regression analyses showed that the effect of deployment trauma on PTSD was dependent on COMT polymorphism with carriers of the homozygous genotypes (Met/Met and Val/Val) showing more PTSD symptoms than those carrying the heterozygous (Val/Met) genotype. This is in line with previous human and animal studies which highlight the role of the Met/Met genotype and show some preliminary support for the Val/Val genotype as a risk factor for the development of PTSD (32–34).

### Epigenetics

Several cross-sectional studies support the putative role of epigenetic mechanisms, especially DNA methylation, in the impact of traumatic stress on mental health (35–38). Recently, prospective epigenetic studies have started to investigate the links between changes in PTSD symptom scores and changes in epigenetic profiles across the period of exposure to traumatic stress. These studies were conducted in subsamples of the PRISMO project and focus on the glucocorticoid receptor exon 1_F_ (GR-1_F_) region and the predictive role of epigenetic markers in PTSD. In the first study, methylation signatures of the GR-1_F_ region (52 loci) were quantified in peripheral blood cells of 92 Dutch military personnel which were collected before and after a 4-month deployment period to Afghanistan. More specifically, the authors focused on mean methylation across all cytosine–phosphate–guanines (CpGs), the number of methylated loci and those CpGs of which methylation was known from previous publications to be associated with GR exon 1_F_ mRNA expression. The latter was termed “functional methylation”. It was found that an increase in either of the methylation levels (i.e., mean, number, and functional) within this region was associated with increases in PTSD symptom scores in trauma-exposed subject. Increased functional methylation was associated with mental health. However, PTSD and mental health problems occurring 6 months post-deployment within individuals exposed to trauma were predicted by neither of the pre-deployment methylation levels (i.e., mean, number, or functional) (21).

In a recent prospective epigenetic study performed using two military cohorts (20), the impact of traumatic stress during combat on post-deployment PTSD symptoms and associated longitudinal epigenetic changes was investigated. In a discovery sample of 93 male Dutch servicemen [PRISMO cohort; same subjects as Schur et al. (21)], specific DNA methylation alterations were associated with the development of PTSD. This cohort displayed changes at 17 positions and 12 regions and subsequent bioinformatic analyses highlighted the role of different pathways linked to PTSD symptomatology. Interestingly, the associations between the development of PTSD symptoms and decreased DNA methylation at genomic regions in *ZFP57, RNF39*, and *HIST1H2APS2* were replicated in a male US marine cohort of MRS with a 7-month war-zone deployment to Iraq or Afghanistan (*n* = 98).

It is worth mentioning that international efforts such as the Psychiatric Genomics Consortium (PGC) PTSD group, which includes data on a large combined sample of four military studies and three civilian cohorts (*N* = 1,147), might have increased statistical power to detect further relevant epigenetic variations and thereby provide deeper insights in the near future (39).

### Blood Markers

#### Inflammatory Markers

Upon the observation that PTSD co-occurred with peripheral inflammation in cross-sectional studies, the question arose as to whether inflammatory markers are causally involved in the disorder or are one of its consequences (40). Since then, several prospective studies have attempted to evaluate the causal role of various inflammatory responses in the development of PTSD.

One study used a subset of the MRS dataset (*N* = 1,719) and found that in U.S. Marines, baseline plasma levels of C-reactive protein (CRP) was a strong predictor of post-deployment PTSD symptoms (22). Another study analyzed gene co-expression profiles obtained through RNA sequencing of peripheral blood leukocytes from Marines belonging to the MRS II (*N* = 124) and replicated the obtained findings in a separate subsample of the MRS (*N* = 50). It was found that both at pre- and post-deployment, co-expression gene networks linked to the innate immune responses, interferon signaling, and monocyte specificity were predictive of post-deployment PTSD (23). Following this and using the same sample as Breen et al. (23), researchers (24) aimed to build upon these findings and ascertained several master regulators driving the previously identified networks. Using ARACNe (Algorithm for Reconstruction of Accurate Cellular Networks) and protein activity analysis they identified SOX3, TNFAIP3, TRAFD1, POU3F3, STAT2, and PML as important master regulators. Gene Ontology analyses enriched by TNFAIP3, TRAFD1, and PML again pointed toward the role of the innate immune responses in the development of PTSD.

In a subsample of the PRISMO dataset (*N* = 693), researchers addressed the immune activation by measuring *in vitro* cytokine production by leukocytes upon stimulation (18). Among other findings, the authors observed a three-way interaction between cytokine production at 1-month post-deployment, trauma exposure during combat (assessed 1-month post-deployment), and post-deployment stressful life events during 12 months post-deployment on the longitudinal changes in PTSD symptoms scores as measured between 1 month and 2 years post deployment. More specifically, increased mitogen-stimulated T-cell and innate cytokine production, greater exposure to stress during combat and during the 12-month post-deployment period were associated with increased PTSD symptoms between 1 month and 2 years post-deployment.

#### Hormonal Dysregulations

Another line of studies focused on the functioning of the hypothalamic–pituitary–adrenal (HPA) axis. In this regard, three studies investigated the links between cortisol levels and stress exposure on the development of PTSD. Two of these studies were part of the PID-PTSD^+3^ project and assessed hair cortisol concentration (HCC) and cortisol stress reactivity, measured through saliva cortisol levels before and after the Trier Social Stress Test (TSST), prior to and following a deployment period of ~5 months (25, 26). Their main finding showed that when exposed to trauma, a lower baseline HCC and lower cortisol stress level were predictive of higher post-deployment PTSD symptomatology (25) while lower HCC predicted higher daily alcohol consumption (26). In another study done in the PRISMO cohort (N = 455), plasma cortisol levels at baseline did not predict PTSD status 6 months after a 4 months deployment period (17). Next to cortisol, these researchers further investigated other crucial molecules of the HPA axis. van Zuiden et al. evaluated the predictive role of mRNA expression of GR–α, GR-P, GR-β, glucocorticoid- induced leucine zipper (*GILZ*), glucocorticoid-inducible kinase-1 (*SGK-1*), or *FKBP5* in peripheral blood mononuclear cells (PBMCs) and the number of GRs in PBMCs on post-deployment PTSD status. Interestingly, only the number of GRs in PBMCs predicted post-deployment PTSD status (17).

Other researchers assessed whether plasma oxytocin (pOT) and arginine vasopressin (pAVP) levels could be used as biomarkers for stress-related development of PTSD (19) in PRISMO. By investigating a group of 907 military subjects, no effects of pOT and pAVP on post-deployment PTSD was observed (19).

Together, these studies highlight the value of prospective studies in linking circulating markers with the development of PTSD. While the first line of evidence suggests that elements of the immune system emerge as candidate biomarkers, there is apparent need for replications and larger longitudinal studies to confirm and extend these initial findings.

## Promoting Resilience

The previous sections provided an overview of prospective human and animal studies that aimed at gaining knowledge of the mechanisms underlying mental illness and resilience. Research in this field has also turned toward studying strategies which could potentially promote mental health and boost resilience. As postulated by McEwen et al., the notion that the brain holds the ability to successfully adapt to changing environments throughout the life course, encourages one to develop top-down interventions encompassing mind-body interactions in order to install fundamental changes in various aspects of one's sense of well-being (41). Given the previously mentioned need to expand alternative add-on strategies in order to promote resilience in today's society, this section will cover a range of psychological, behavioral and lifestyle interventions which aim to do so.

### Mindfulness and Meditation

Over the last few decades, meditation techniques such as loving-kindness meditation and mindfulness meditation have been spreading in the western world. Today, mindfulness mainly owes its popularity to Professor Jon Kabat-Zinn who reintroduced it in his mindfulness-based stress reduction (MBSR) program. Described as the awareness that arises through paying purposeful and non-judgemental attention to the present moment (42), mindfulness is now employed as part of standardized programs aiming to promote general human well-being and install deeply rooted positive emotions.

Empirical evidences about the benefits of mindfulness-based programs are inciting a growing interest in the (neuro)biological underpinnings of mindfulness. Different mindfulness programs have shown to impact both gray and white matter density of several brain structures such as in the right basolateral amygdala (43) and bilateral clusters within the brainstem including the pontine tegmentum, locus coeruleus, nucleus raphe, and the sensory trigeminal nucleus (44). Moreover, findings show that other types of mind-body interventions also influence various parameters of the immune system. For example, one study showed that following a yogic meditation, the activity of the proinflammatory nuclear factor-kappa beta (NF-κB), known to have a prominent role in inflammation and stress, was reduced in peripheral blood leukocytes as compared to baseline measures (45). Another study found an increase in telomerase activity along with reduced levels of another inflammatory marker, CRP, in peripheral blood mononuclear cells (PBMCs) following the same type of meditation (46). Yet another study found that by measuring gene expression in peripheral blood prior to and following a deep relaxation session, the expression of genes associated with telomere maintenance were enhanced at follow-up while specific genes linked to stress-related pathways were reduced in expression (47). Although interesting, it is important to note that currently performed studies vary greatly in terms of the type of (mindfulness) meditation used along with the research designs and (often small) sample sizes. Gaining knowledge in the mechanisms underlying the well-documented stress-reducing and mood-enhancing effects of meditation and mindfulness-based programs (48–51) holds the potential to further help the design of powerful strategies in healthcare settings to promote the cultivation of a healthy mind.

### Cognitive Behavioral Therapy-Based Programs

Cognitive behavioral therapy (CBT) was originally developed by Aaron Beck to promote mental wellness and coping resources in patients suffering from mental distress such as depression, anxiety and chronic stress (52). The idea of CBT is to modify one's thinking and behavioral patterns which color the way life events are being experienced. Interestingly, combining CBT with pharmacological treatments such as cognitive enhancers (but not anxiolytics) has been shown to improve long-term treatment efficacy and fear extinction, potentially by enhancing memory consolidation [recently extensively reviewed in Singewald et al. (53)]. In addition, conducting CBT sessions such as exposure therapy before sleep has also been suggested to enhance treatment efficacy, raising the question whether pharmacological approaches can be implemented to enhance memory consolidations during sleep specifically (53, 54). Another intervention targeting memory consolidation involves playing a computer game with high visuospatial demands (e.g., Tetris) during the hours following a traumatic event. Interestingly, this approach has recently been suggested to disrupt the consolidation of trauma memories and lead to fewer intrusive visual memories of the traumatic event (55). In order to maximize treatment outcome, combinations of different behavioral approaches with or without various pharmacological options will need further testing.

Besides its well-documented therapeutic effect in treating mental distress and some other disorders, today's interest in CBT is also geared toward the construction of a personal model to boost resilience in the face of life's obstacles without necessarily targeting a particular mental disorder. Padesky and Mooney have proposed a CBT-based program entirely oriented toward “resiliency” research (56). Their so-called strengths-based CBT consists of four sessions in which the therapist and the client actively collaborate to explore and reinforce positive qualities such as interpersonal competences, self-efficacy or self-esteem. The therapist and client co-create a personal model of resiliency by turning the previously identified strengths into effective strategies that can be applied in everyday situations. Another CBT-based program is the Stress Inoculation Therapy (SIT) which was first introduced in 1985 (57). In social psychology, the concept of inoculation refers to the preventive effect of brief and moderately challenging stress exposures on one's reaction to subsequent, more intense stressors. More specifically, exposing animals or individuals to minor stressors has been shown to enhance one's resilience or “inoculate” them from later stressful situations (58–60). SIT incorporates this notion of inoculation in a psychotherapeutic intervention that combines cognitive and behavioral methods emphasizing coping skills learning. During a SIT session, an individual is exposed to and learns to cope with increasing amounts of stress through productive thoughts, mental images, self-statements, and relaxation training thereby enhancing his or her immunity to stress. Empirical evidence shows that SIT efficiently reduces stress, anxiety and depression in cancer patients (61) and effectively reduces psychological distress up to 3 months following the sessions when delivered through two half-day training sessions in the workplace (62).

### Physical Activity

It is commonly known that practicing regular physical exercise leads to a plethora of positive health effects (63). These benefits not only include cardiovascular and metabolic effects, but also improvements in cognitive abilities. Previous studies on animals and humans have revealed increases in synaptic plasticity and neurogenesis (64), strengthened cortical activation when performing a cognitively challenging task (65) and improvements in learning, slowing the course of cognitive decline in aging (65, 66). Using a within-subject design and multiple momentary assessments collected through experience sampling method (ESM), a diary technique assessing daily moods and activities, one study provides support for a causal effect of physical activity on positive affect (67). Since the extent of positive affect levels varied between individuals based on their history of clinical depression, such findings call for individually tailored interventions in which clinicians could adapt the amount of physical exercise. Other studies showed that compared to training exercises with no cognitive component, specific exercises that promote mindfulness by means of calming techniques and cognitive strategies such as yoga or pilates were more effective in eliciting psychological benefits such as mood enhancements and improved executive functions over time (68–70). Mindful-based physical activities, thus, seem to help improve breathing rate and depth (71, 72) along with heart rate (72) while lowering arousal levels (73). Further comparative trials in populations at high risk of robust exposure to traumatic stress are needed in order to prospectively assess the putative protective properties of these interventions on trauma-related mental ill-health.

### Social Support

Several lines of evidence confirm the importance of pursuing cognitive and social activities to maintain global mental and functional health (74–76). However, the exact biopsychological mechanisms underlying the positive impact of social support on mental well-being and resilience to stress still remain unclear (77). To enhance both cognitive and social aspects, programs such as the Experience Corps have been introduced. This intergenerational program was originally designed by Fried et al. in 1997 to promote health among the aging population. Specifically, this program encourages adults over the age of 50 to share their skills with children needing help at school. While students obtain greater academic outcomes, older adults get the opportunity to enrich their lives on a social and cognitive level (78). A recent study shows that this program further significantly slows the normal age-related decrease in cortical and hippocampal brain volumes (79).

### Meaning and Purpose in Life

Programs such as the Experience Corps offer older adults a sense of meaning and purpose in life which is a crucial component of mental health. A meta-analysis found that purpose in life was strongly linked to social integration in a population of older individuals and was further related to factors such as quality of life, a better health, and socioeconomic status (80). Other studies have suggested that finding a sense of purpose in life is a partial mediator of the observed negative association between trait mindfulness and outcomes such as anxiety and depressive symptoms (81). Having meaning to life has further been negatively associated with suicidal ideation (82, 83) while being an important predictor of depressive symptoms (84). Taken together, these findings highlight the importance of establishing personal values and long-term goals in order to help one overcome or prevent psychological problems.

## Discussion

As reflected by the first part of this review, there is considerable variation in the way resilience is currently being understood, defined and measured both within animal and human studies.

In animals, the main challenge is to understand how one can identify a “resilient” animal and how this relates to resilience in humans. Most animal studies of resilience identify resilient animals based on the absence of stress-related behavioral features. However, when assessing an animal's behavior, one should evaluate both the absence of stress-related behavioral features or biological markers, and the presence of adaptive behaviors or markers. For an overview of studies that have started to identify such adaptive behavioral, neural and molecular mechanisms, the reader is referred to Pfau and Russo (85) and Russo et al. (86). Furthermore, only few animal studies have incorporated a baseline behavioral or physiological measure in order to assess dynamic changes over time. Using cross-sectional designs, recent studies identified distinct molecular signatures that were associated with adaptive or maladaptive behavioral responses to stress (87–89). Adding a baseline measurement to such approaches would be highly valuable in identifying baseline differences between animals along with the pattern of change from pre- to post-stressor within the same animal. Linking such markers with differential susceptibility to a stressor will further yield valuable insights into the molecular mechanisms of resilience which, in turn, will more efficiently lead to the identification of (a combination of) predictive biomarkers of resilience. Lastly, researchers need to carefully reflect upon their animal model (e.g., sex, strain, and age), phenotypes of interest and experimental designs (e.g., timing, duration, and type of stressor) (90) in order to fulfill different types of validities to make their tests and models translatable to the clinical situation (91, 92).

In humans, although conducting prospective studies has received increasing attention, most findings are still preliminary since replication is often lacking and most studies harbor low effect sizes and relatively small sample sizes. It is also important to note that (i) a wide variety of tools (which cannot be compared easily) are currently being used to measure “resilience” of which the validity needs critical examination, (ii) the majority of resilience studies focuses on PTSD-related outcomes instead of positive outcome measures, and (iii) most of the studies which fulfilled the rather strict inclusion and exclusion criteria were conducted in military cohorts. While such samples provide a unique opportunity to study the effects of trauma exposure, they are also subjected to a natural limitation since sampling bias cannot be excluded.

For future studies, researchers are encouraged to include a wider range of assessments when aiming to study and measure resilience in order to obtain a more reliable and objective operationalization of this concept. Using several techniques such as ESM (93), in-person interviews combined with self-evaluations or targeted questionnaires and physiological measures including heart rate and blood pressure will allow one to obtain a more global picture on general psychological and physiological health. Furthermore, ESM might help to better understand processes such as inoculation during which individuals may develop resilience through repeated stress exposure. When possible, this, again, should be embedded within large-scale longitudinal studies since these allow tracking the stability of one's mental health over a specific time period. Moreover, and in order to facilitate extrapolation to the general population, there is a strong need for the inclusion of women in these studies, which is currently underdone (94). This is crucial when knowing that women are more likely than men to develop stress-related mental disorders. Although this is a much broader phenomenon within science, both in animal and human research, women are even less likely to be included in studies using military cohorts in which they are underrepresented. An increase in the number of studies focusing on this population will undoubtedly enhance our current understanding of their stress responsiveness, health care and gender-specific needs. Lastly, in the context of searching for biological underpinnings or biomarkers that reliably predict differential susceptibility to future stress or psychological and biological resilience mechanisms, exploring the potential to combine several predictors, e.g., genetic, epigenetic, and/or imaging data on an individual basis is strongly encouraged. This further calls for more large-scale brain imaging studies in order to identify the brain regions involved in stress resilience.

Finally, establishing alternative strategies aiming to install positive emotions and improve cognitive abilities, social interactions, feelings of purpose and meaning of life along with physical health have obtained scientific evidence for their benefits in increasing one's global mental health, whilst the biological underpinning of these effects have remained understudied thus far. Gaining a deeper understanding of the underlying mechanisms of each of these strategies will undoubtedly aid in developing new treatment options for stress-related disorders such as PTSD.

## Author Contributions

CS and L-KP contributed equally to this manuscript. GAJ contributed to the interpretation of the obtained data. NS, GAJ, NY, LdN, and SG reviewed the manuscript and provided comments/suggestions. BR reviewed the manuscript, provided comments/suggestions, and is the corresponding author.

### Conflict of Interest Statement

The authors declare that the research was conducted in the absence of any commercial or financial relationships that could be construed as a potential conflict of interest.
